# Strengthening tuberculosis control: addressing gaps in screening, diagnosis, and funding

**DOI:** 10.1016/j.ijregi.2025.100650

**Published:** 2025-04-12

**Authors:** Harsh Shah, Jay Patel, Sandeeep Rai, Abhishek Sen

**Affiliations:** 1 Indian Institute of Public Health Gandhinagar (IIPHG), Gandhinagar, India

Tuberculosis (TB) remains a major public health challenge, particularly, in high-burden settings such as India. Although progress has been made in screening household contacts of index patients with TB, limitations in current diagnostic approaches continue to hinder comprehensive detection. In 2022, India reported screening 94% (3.16 million) of household contacts, surpassing the global average of 84% [1]. However, standard symptom-based screening tools may miss a significant proportion of asymptomatic or pauci-symptomatic cases, necessitating improved detection strategies. Integrating artificial intelligence–based computer-aided diagnosis with chest X-rays and World Health Organization–approved rapid molecular diagnostics can enhance case detection and treatment initiation [2].

A critical concern is the persistent global and India-specific gap in TB case detection. Approximately 4 million people with TB remain undiagnosed or unreported annually, exacerbating transmission risks [3]. This gap is influenced by challenges in index case identification, barriers in diagnostic access, and social stigma. Addressing these obstacles through expanded community engagement and innovative screening techniques is essential to bridging this detection deficit and ensuring equitable access to TB preventive treatment [4].

Financial constraints further impede TB elimination efforts. In 2023, funding for TB prevention, diagnosis, and treatment in low- and middle-income countries, including India, totaled US$ 5.7 billion, with India’s domestic contribution comprising only a fraction of this [5]. The withdrawal of United States Agency for International Development (USAID) funding has further strained resources, raising concerns about the sustainability of TB programs. Without increased investment, an estimated 2.2 million TB-related deaths and 10.6 million new cases could occur by 2030 [6]. Strengthened financial commitments from domestic and international sources are critical to sustaining and accelerating TB control initiatives.

Addressing these gaps in screening, diagnosis, and funding is vital to achieving TB elimination. A concerted effort is required to enhance early case detection, improve access to TB preventive treatment, and secure adequate financial resources to sustain long-term TB control measures.

## Declarations of competing interest

The authors have no competing interests to declare.
